# Distinct *In Vitro* T-Helper 17 Differentiation Capacity of Peripheral Naive T Cells in Rheumatoid and Psoriatic Arthritis

**DOI:** 10.3389/fimmu.2018.00606

**Published:** 2018-04-04

**Authors:** Eszter Baricza, Nikolett Marton, Panna Királyhidi, Orsolya Tünde Kovács, Ilona Kovácsné Székely, Eszter Lajkó, Lászó Kőhidai, Bernadett Rojkovich, Barbara Érsek, Edit Irén Buzás, György Nagy

**Affiliations:** ^1^Department of Genetics, Cell- and Immunobiology, Semmelweis University, Budapest, Hungary; ^2^Department of Methodology, Budapest Business School, Budapest, Hungary; ^3^Buda Hospital of the Hospitaller Order of Saint John of God, Budapest, Hungary; ^4^Office for Research Groups Attached to Universities and Other Institutions of the Hungarian Academy of Sciences, Budapest, Hungary; ^5^MTA-SE Immune-Proteogenomics Extracellular Vesicle Research Group, Budapest, Hungary; ^6^Department of Rheumatology, 3rd Department of Internal Medicine, Semmelweis University, Budapest, Hungary

**Keywords:** T-helper 17 differentiation, rheumatoid arthritis, psoriatic arthritis, interleukin-17A, interleukin-22

## Abstract

**Background:**

The T-helper 17 (Th17) cells have a prominent role in inflammation as well as in bone and join destruction in both rheumatoid and psoriatic arthritis (RA and PsA). Here, we studied Th17 cell differentiation in RA and PsA.

**Methods:**

Blood samples from healthy donors, RA and PsA patients were collected. CD45RO^−^ (naive) and CD45RO^+^ (memory) T cells were isolated from peripherial blood mononuclear cell by magnetic separation. Naive T cells were stimulated with anti-CD3, anti-CD28, and goat anti-mouse IgG antibodies and treated with transforming grow factor beta, interleukin (IL)-6, IL-1_β_, and IL-23 cytokines and also with anti-IL-4 antibody. IL-17A and IL-22 production were measured by enzyme linked immunosorbent assay, *RORC*, and *T-box 21* (*TBX21*) expression were analyzed by quantitative polymerase chain reaction and flow cytometry. C-C chemokine receptor 6 (CCR6), CCR4, and C-X-C motif chemokine receptor 3 expression were determined by flow cytometry. Cell viability was monitored by impedance-based cell analyzer (CASY-TT).

**Results:**

*RORC, TBX21*, CCR6, and CCR4 expression of memory T cells of healthy individuals (but not RA or PsA patients) were increased (*p* < 0.01; *p* < 0.001; *p* < 0.05; *p* < 0.05, respectively) compared to the naive cells. Cytokine-induced IL-17A production was different in both RA and PsA patients when compared to healthy donors (*p* = 0.0000026 and *p* = 0.0001047, respectively). By contrast, significant differences in IL-22 production were observed only between RA versus healthy or RA versus PsA patients (*p* = 0.000006; *p* = 0.0013454, respectively), but not between healthy donors versus PsA patients.

**Conclusion:**

The naive CD4 T-lymphocytes are predisposed to differentiate into Th17 cells and the *in vitro* Th17 cell differentiation is profoundly altered in both RA and PsA.

## Introduction

T-helper 17 (Th17) cells are the third major subpopulation in addition to Th1 and Th2, which was described in mice (1) and in human (2). They are the main producers of interleukin-17A (IL-17A), IL-17F, IL-22, and tumor necrosis factor alpha (TNF_α_) (2–6). Th17 cells are necessary for the physiological defense against extracellular bacterial and fungal infections. In addition, they contribute to inflammation in several autoimmune diseases, such as rheumatoid arthritis (RA), systemic lupus erythematosus (SLE), multiple sclerosis, inflammatory bowel diseases, psoriasis, or psoriatic arthritis (PsA) (3–8).

The Th17-derived cytokines induce the production of numerous inflammatory mediator and effector molecules, such as proinflammatory cytokines IL-6 and IL-1_β_, nitric oxide, matrix metalloproteinases, granulocyte-monocyte colony-stimulating factor (GM-CSF), and granulocyte colony-stimulating factor (G-CSF). This complex effect includes neutrophil recruitment, osteoclastogenesis, synovial proliferation, and finally may lead to bone and joint destruction in RA (3–5, 7, 9–12). IL-17A and IL-22 are co-expressed by Th17 cells and contribute to the pathologic events for example development of the psoriatic plaque, pannus formation in the joint, joint erosion, and new bone formation in PsA (3–7, 9–18).

Human IL-17A producing cells originate from cluster of differentiation 161 (CD161)^+^CD4^+^ progenitor cells, which constitutively express retinoic acid-related orphan receptor variant 2 (*RORC2*) (19–21). *RORC2 gene* encodes the RAR-related orphan receptor gamma (RORγ) which is a master regulator of human Th17 cells (20, 22). The Th1-specific transcription factor, T-box 21 (*TBX21*) encodes for the T cell-specific T-box transcription factor T-bet (T-bet) that regulates interferon gamma (IFN_γ_) production (23). Those IL-17A-producing cells that do not produce IFN_γ_, are characterized by the co-expression of C-C chemokine receptor 6 (CCR6) and CCR4, while CCR6^+^ and C-X-C motif chemokine receptor 3 (CXCR3^+^) cells can produce IL-17A with or without IFN_γ_ (2, 24). Naive CD4^+^ T cells may differentiate toward Th17 cells depending on the initial effect of cytokines, such as transforming grow factor beta (TGF_β_), IL-1_β_, IL-6, IL-23 (2, 22, 25–30), and other regulating factors such as aryl hydrocarbon receptor (AHR) (31).

Interleukin-17A and IL-22 positive cells are highly represented in peripherial blood mononuclear cells (PBMCs) and synovial tissues of RA and in the psoriatic lesions of PsA patients. Furthermore, some studies showed a correlation between the Th17-related cytokine production and the clinical parameters in both diseases. IL-17A targeting biologicals are used in the clinic, while IL-22 is a potential therapeutic target in psoriasis and in inflammatory arthropathies (9–11, 13–17, 32–35).

In this study, we characterized the naive CD4^+^ T cells in healthy individuals and patients with RA and PsA based on their expression of transcription factors and chemokine receptors as well as on their cytokine production during Th17 differentiation.

## Patients and Methods

### Patients

Patients were recruited in the rheumatology outpatient department of the Semmelweis University (Hospital of Hospitaller Brothers of St. John of God, Budapest, Hungary). Patients were diagnosed with RA (*n* = 12) according to the 2010 American College of Rheumatology/European League Against Rheumatism classification criteria (36), or with PsA (*n* = 7) (37); all patients had moderate or active disease. The clinical parameters and treatments of RA and PsA patients were explained in the Table S1 in Supplementary Material. Matched control samples were collected from healthy volunteers (*n* = 12).

### Cell Preparation

Peripheral blood samples were collected into EDTA vacutainer tubes (Greiner Bio-One). PBMCs were isolated from the blood of healthy volunteers, RA, and PsA patients by density gradient centrifugation over Histopaque-1077 (Sigma-Aldrich). For quantitative real-time PCR analysis, the CD4^+^ T cells were negatively isolated from PBMCs with a magnetic-activated cell sorting CD4^+^ T Cell Isolation Kit (Miltenyi Biotec), according to the manufacturer’s instructions. The CD45RO^−^ (negative fractions) and CD45RO^+^ (positive fractions) cells were separated also with a CD45RO Microbeads (Miltenyi Biotec). For flow cytometric analysis of chemokine receptors, the CD4^+^CD45RO^−^ cells were negatively isolated from PBMCs with naive CD4^+^ T Cell isolation kit (Miltenyi Biotec) according to the manufacturer’s instructions. The purity of the separated cells was at least 95% in all cases, as determined by flow cytometry.

### *In Vitro* Cell Culture

The cells were cultured (10^6^/mL) in Roswell Park Memorial Institute 1640 (Sigma-Aldrich) supplemented with 10% fetal bovine serum (Gibco), 2 mM glutamine, and 1% penicillin–streptomycin solution (Sigma). The cells were stimulated with anti-CD3 (1 µg/mL) (R&D Systems), anti-CD28 (1 µg/mL) (BioLegend), and with F(ab′)2 fragment goat anti-mouse IgG (CAB) (1 µg/mL) (Jackson ImmunoResearch) antibodies, and treated with TGF_β_ (2.5 ng/mL), IL-6 (25 ng/mL), IL-1_β_ (10 ng/mL), and IL-23 (10 ng/mL) cytokines (ImmunoTools GmbH), and with anti-IL-4 neutralizing antibody (10 µg/mL) (BioLegend). The following cytokine combination was used to promote Th17 cell differentiation: TGF_β_ + IL-6, TGF_β_ + IL-6 + IL-1_β_, IL-1_β_ + IL-23, and IL-1_β_ + IL-23 + IL-6. Anti-IL4 antibody was used in all cytokine combination treatments to block Th2 development (based on a study reported by Bettelli et al. (25) and our unpublished data). Fifty percent of cell supernatants were collected on the fifth day of differentiation and the same volume was added, supplemented with the appropriate cytokines. The cells were treated for 10 days and different samples were collected initially then on the 5th and 10th days for analysis. Cell viability was monitored by an impendance-based cell analyzer (CASY-TT) (Roche Innovatis AG).

### Quantitative Real-Time PCR

Total RNA was isolated with NucleoSpin RNA/Protein kit (Macherey-Nagel) and the quantity of RNA was determined by NanoDrop ND-1000 spectrophotometer (NanoDrop Technologies). The total amount of RNA was 1,000–4,000 ng/sample, which was isolated from 20,000 to 40,000 cells (there was no significant difference between samples from patients and controls). Complementary deoxyribonucleic acid (cDNA) was synthesized from total amount of RNA with a SensiFAST cDNA Synthesis Kit (Bioline) in accordance with the manufacturer’s instructions. The real-time PCRs were carried out in PCR Master Mix containing SensiFAST™ Probe Hi-ROX Kit (Bioline) using TaqMan assays (Thermo Fisher Scientific) for hypoxanthine phosphoribosyltransferase 1 (*HPRT-1*) (Hs02800695_m1) or *RORC* (Hs01076122_m1) or *TBX21* (Hs00203436_m1) and 25 ng cDNA per gene/well in 8 µL final volume. Specific transcript levels were referred to those of HPRT-1; and the ΔΔC_t_ calculation method was used to determine the appropriate gene expressions (38).

### Enzyme Linked Immunosorbent Assay (ELISA)

Interleukin-17A and IL-22 levels were measured by human IL-17A and IL-22 ELISA Ready-SET-Go kits (eBioscience), according to the manufacturer’s protocol with the appropriate standards.

### Flow Cytometry

C-C chemokine receptor CCR6, CCR4, and CXCR3 expression of the freshly separated CD4^+^CD45RO^−^, CD4^+^CD45RO^+^, and the differentiated cells were measured by flow cytometry. The cells were centrifuged and stained in PBS containing 0.5% BSA for 30 min at 4°C with anti-human CCR6 FITC (BioLegend), anti-human CCR4 PE (BioLegend), anti-human CXCR3 PerCP Cy5.5 (BioLegend), and anti-human CD4 APC (BD Biosciences) antibodies or with the appropriate isotype controls. After washing, 5 × 10^4^ cells were measured with fluorescence-activated cell sorting (FACS) Calibur flow cytometer (BD Biosciences). Data were analyzed with FlowJo (Tree Star, Ashland, OR, USA). To determine the RORγ and T-bet expression of naive, central and effector memory cells, the freshly isolated PBMCs were permeabilized and fixed using transcription buffer set (BD Biosciences) according to the manufacturer’s instructions. The samples were stained with human naive/memory T cell ID panel antibody (containing anti-human CD3 APC/Cy7, anti-human CD4 PerCP Cy5.5, anti-human CD45RA FITC, and anti-human CD197 APC) (BioLegend), anti-human CD45RO PE/Cy7 (BioLegend), anti-human T-bet PE CF597 (BD Biosciences), and anti-human RORγ PE (BD Biosciences) antibodies. After washing, 5 × 10^5^ cells were measured with FACS Calibur flow cytometer (BD Biosciences), analyzed with FlowJo (Tree Star, Ashland, OR, USA). Gating strategy was shown in Figure S1 in Supplementary Material.

### Statistical Analysis

GraphPad Prism Software V6 (San Diego, CA, USA) was used for univariate statistical analyses. For the comparison of two dependent groups the Wilcoxon matched-pairs signed rank test, for two independent groups’ comparison the Mann–Whitney test, and for multiple group comparison the Friedman test was used. In case, significant differences were observed for the pair wise analysis, the Wilcoxon matched-pairs signed rank test or the Mann–Whitney test was also applied after the Friedman test. *p*-Values below 0.05 were considered as statistically significant. Correlation analysis was used to examine linear correlation between data. Using the R statistical software (39), analysis of variance (40) and consecutively Tukey HSD tests (41) were carried out to investigate the differences in the cytokine treatment-induced IL-17A and IL-22 production between healthy, RA, and PsA groups. Linear discriminant analysis (LDA) was used to compare and classify the healthy, RA, and PsA individuals based on the data regarding transcription factors, cytokine production, and chemokine receptor expression (42). The detailed description of the LDA method was summarized in the Data Sheet S1 in Supplementary Material.

## Results

### The Naive T Cells of RA and PsA Patients Are Committed Toward Th17 and to a Lesser Extent Toward Th1 Cells Based on Their *RORC* and *TBX21* Expression

**Figure 1:**
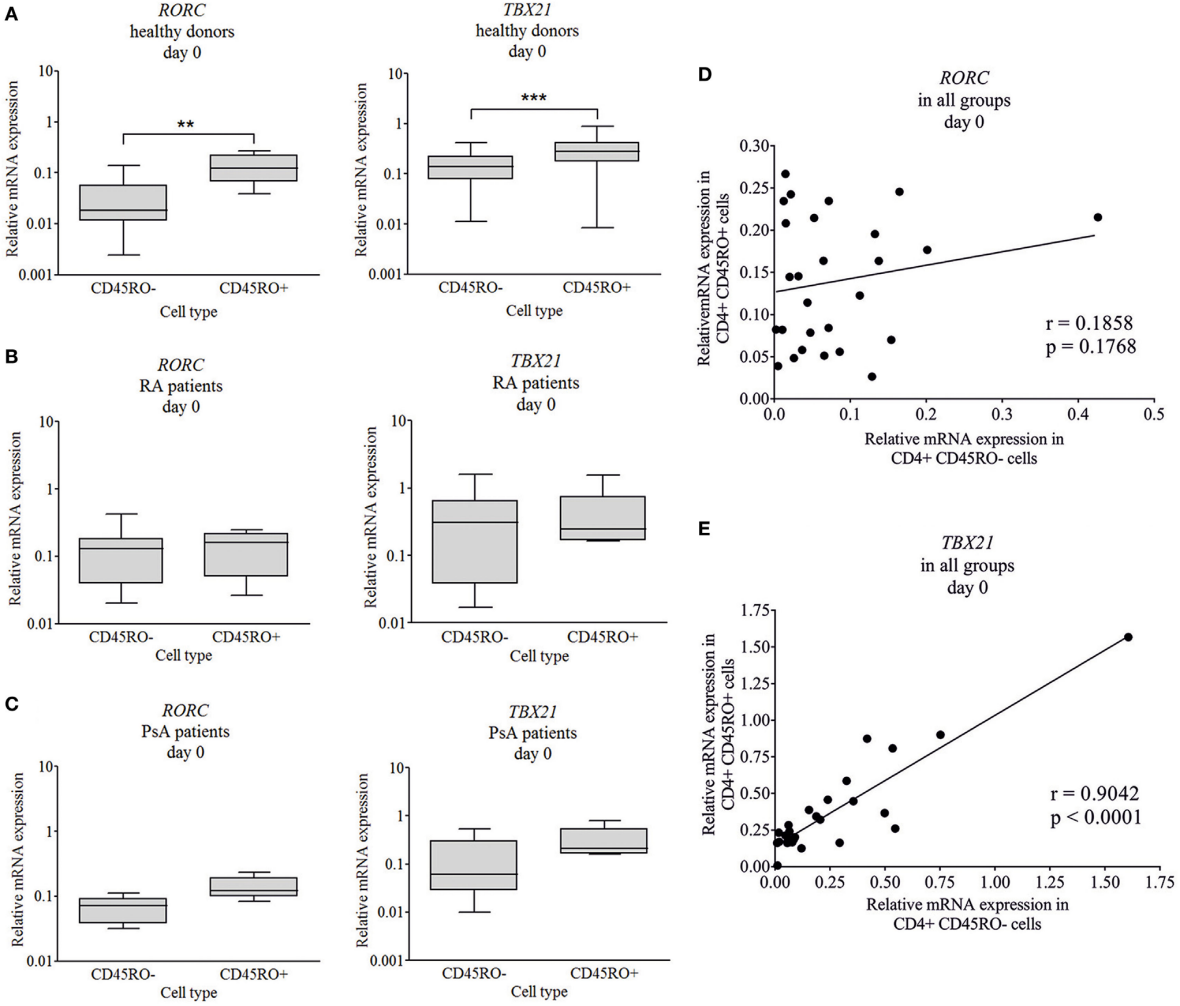
Baseline *RORC* and *TBX21* transcription factor expression in naive and memory cells. CD4^+^CD45RO^−^ naive and CD4^+^CD45RO^+^ memory T cells were isolated by magnetic separation from peripherial blood mononuclear cells of healthy volunteers (*n* = 11), rheumatoid arthritis (RA) (*n* = 10), and psoriatic arthritis (PsA) patients (*n* = 5). Total RNA was isolated and the gene expressions were measured by quantitative real-time PCR. Healthy donors’ **(A)**, RA **(B)**, and PsA patients’ **(C)** data are shown in a logarithmic scale; median, minimum, and maximum of the relative gene expression levels are indicated (Wilcoxon signed rank test **p < 0.01; ***p < 0.001). Correlations (Pearson correlation) are shown between the *RORC* **(D)** and *TBX21* **(E)** expression in naive and memory T cells.

To characterize CD4 plasticity, master regulators of the Th17 and Th1 engagement were measured. The CD4^+^CD45RO^+^ (memory Th cells/Tm) and CD4^+^ CD45RO^−^ (naive Th cells) cells were isolated from healthy donors, RA, and PsA patients and Th17- and Th1-specific transcription factors were measured by quantitative real-time PCR. In healthy donors, an increased messenger ribonucleic acid (mRNA) expression of both the *RORC* and *TBX21* transcription factors were detected (6.6-fold increase, *p* < 0.01 and 2-fold increase, *p* < 0.0001, respectively) in memory cells, as compared to naive cells (Figure 1A). By contrast, no significant difference was observed in the *RORC* and *TBX21* expression between the CD45RO^+^ and CD45RO^−^ cells neither in RA nor in PsA (Figures 1B,C).

**Figure 2:**
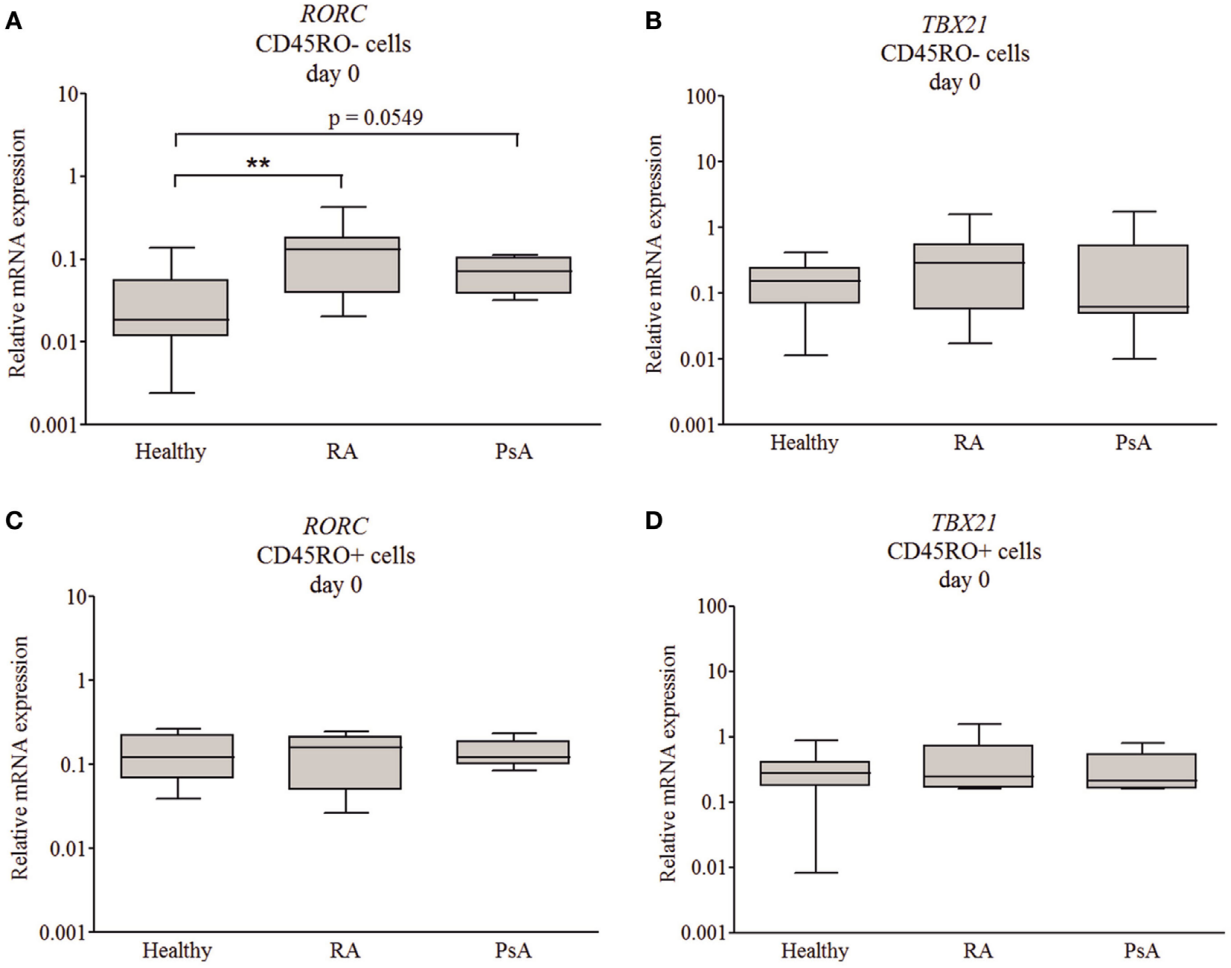

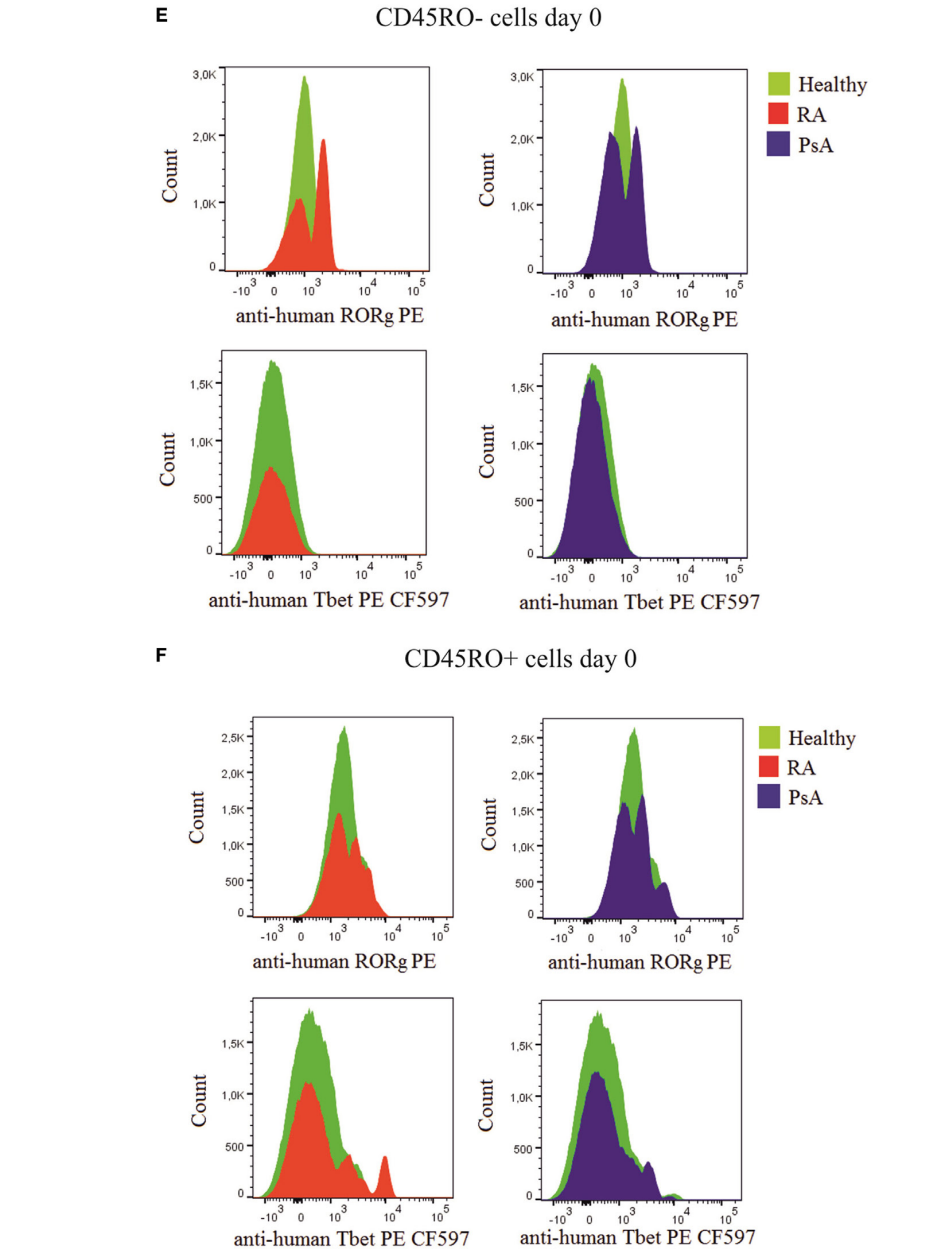
*RORC* and *TBX21* expression across patient and healthy donor groups. Messenger ribonucleic acid (mRNA) expressions of *RORC* and *TBX21* in naive **(A,B)** and memory **(C,D)** T cells. Gene expressions were measured by quantitative real-time PCR; the values are shown in a logarithmic scale; median, minimum, and maximum of the relative gene expression levels are indicated (Mann–Whitney test **p < 0.01). Flow cytometry was used to study the RAR-related orphan receptor gamma (RORγ) and T cell-specific T-box transcription factor T-bet (T-bet) protein levels of individual cells from healthy donors (*n* = 5), rheumatoid arthritis (RA) (*n* = 5), and psoriatic arthritis (PsA) (*n* = 5) patients. Cumulative histograms of the naive **(E)** and memory **(F)** T lymphocytes are shown.

Interestingly, while the *TBX21* expression of the naive and memory T cells correlated strongly (Figure 1E, *r* = 0.9; *p* < 0.0001), the *RORC* expression did not show any correlation (Figure 1D, *r* = 0.18; *p* = 0.17) between the two CD4^+^ T cell subsets. These correlations (Pearson) support our main findings, regarding the increased *RORC* expression (Figure 2A; *p* < 0.01 and *p* = 0.054, respectively) of naive T cells in RA and PsA (no correlation Figure 1D), while the *TBX21* expression of the naive cells of all patients and controls is similar (Figure 2B; strong correlation Figure 1E).

Intracellular flow cytometry analysis was used to characterize transcription factor expression levels of the individual cells (Figures 2A–D). The naive (CD3^+^CD4^+^CD45RO^−^CD45RA^+^CD197^+^), effector (CD197^−^/Tem), and central (CD197^+^/Tcm) memory T cells (CD3^+^CD4^+^CD45RO^+^CD45RA^−^) were studied. Although the T-bet expression of the healthy, RA, and PsA-derived naive cells was similar; the RORγ distribution of the RA and PsA samples was apparently sifted, as the cumulative images show (Figures 2E,F). These data confirm the increased RORγ expression of the naive T cells in both RA and PsA. The effector memory cells represent a substantial part of the memory cells (38.8% of the Tm cells) in all groups (without any remarkable difference between the patients and controls). These cells express RORγ or T-bet only or both T-bet and RORγ more frequently than the central memory T cells (Tcm cells represent 61.2% of the Tm cells; *p* = 0.0128, *p* < 0.0001, and *p* < 0.0001, respectively, data not shown).

Linear discriminant analysis was performed to evaluate the data from naive and memory T cell-derived *RORC* and *TBX21* expression. The discriminative power of the healthy, RA, and PsA group separation was 61% and the naive T cells’ *RORC* expression had the strongest determinant role (Figure S2A in Supplementary Material).

### Chemokine Receptor Pattern Confirm the Early Engagement of Naive T Cells

**Figure 3:**
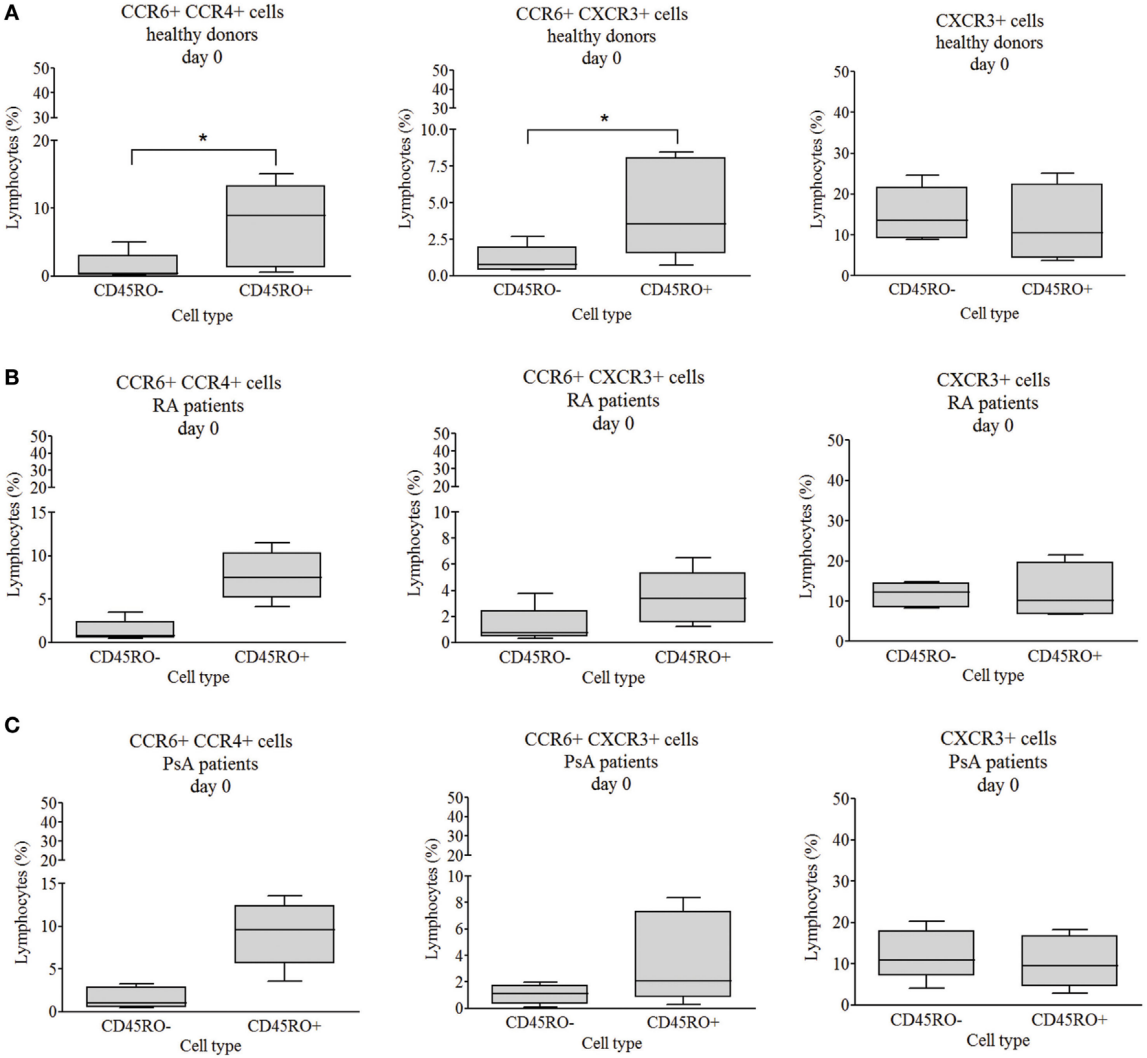
CCR6^+^CCR4^+^, CCR6^+^CXCR3^+^, and CXCR3^+^ chemokine receptor expression. The chemokine receptor expression of CD4^+^CD45RO^−^ naive and CD4^+^CD45RO^+^ memory T cells were studied by flow cytometry. Healthy volunteers’ [**(A)** *n* = 6], rheumatoid arthritis (RA) [**(B)** *n* = 5], and psoriatic arthritis (PsA) patients’ [**(C)** *n* = 5] data. The values are shown in a linear scale; the median, minimum, and maximum values are indicated (Wilcoxon signed rank test *p < 0.05).

To study the phenotype of T cell subsets, CCR4, CCR6, and CXCR3 chemokine receptor expression of isolated CD4^+^ T cells was measured by flow cytometry. Except for CXCR3, all chemokine receptors and their combinations had largely higher expression on the surface of the healthy-derived CD45RO^+^ memory T cells as compared to CD45RO^−^ naive cells (*p* < 0.05 in all cases, Figure 3A; Figure S3A in Supplementary Material). By contrast, there was no significant difference between the chemokine receptor expression of naive and memory cells of either RA- or PsA-derived CD4^+^ T cells (Figures 3B,C; Figures S3B,C in Supplementary Material). These data support the premature engagement of naive T cells in both RA and PsA.

Naive T cells from healthy individuals, RA, and PsA patients were discriminated from each other with 81.3% power by the chemokine receptor expression data and the determinant factor was the proportion of the CCR4^+^CXCR3^+^ memory T cells (Figure S2B in Supplementary Material) by LDA analysis.

### Th17 Cell Differentiation Promoting Factors in RA and PsA

Although there is a general agreement regarding the key conditions initiating the human Th17 cell differentiation, several different cytokine combinations were reported to promote this process (2, 22, 25–30). Based on previous publications and our experience, in addition to CD3/CD28/CAB stimulation (43) and anti-IL4 antibody (25), the following four cytokine combinations were applied to promote naive CD4^+^ T cell differentiation to Th17: TGF_β_ + IL6, TGF_β_ + IL6 + IL1_β_, IL1_β_ + IL23, and IL1_β_ + IL23 + IL6.

**Figure 4:**
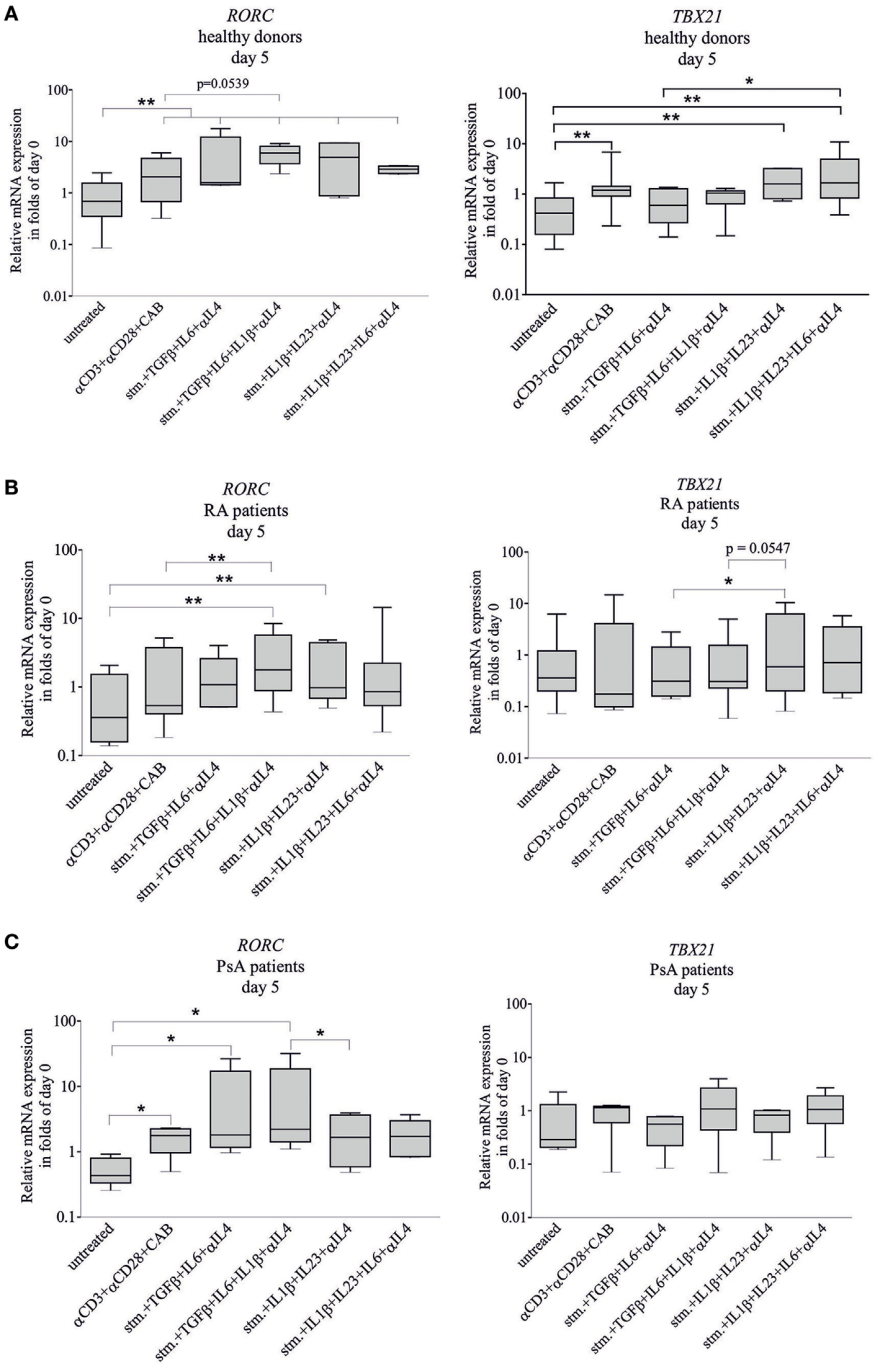
*RORC* and *TBX21* expression during T-helper 17 (Th17) differentiation. Naive CD4^+^ T cells were differentiated toward Th17-lane. Samples were treated with CD3/CD28/CAB (stim) or with stim + anti-IL4 antibody and the following four cytokine combinations to promote naive CD4^+^ T cell differentiation: transforming grow factor beta (TGF_β_) + IL6, TGF_β_ + IL6 + IL1_β_, IL1_β_ + IL23, and IL1_β_ + IL23 + IL6. Total RNA was isolated and the gene expressions were measured by quantitative real-time PCR on the fifth day of the differentiation. *RORC* and *TBX21* transcription factor expressions of cells derived from healthy donors [**(A)** *n* = 10], rheumatoid arthritis (RA) [**(B)** *n* = 10], and psoriatic arthritis (PsA) patients [**(C)** *n* = 6] are shown in a logarithmic scale. Untreated naive cells served as controls. The median, minimum, and maximum values are indicated (Friedman test and pairwise Wilcoxon signed rank test *p < 0.05, **p < 0.01).

**Figure 5:**
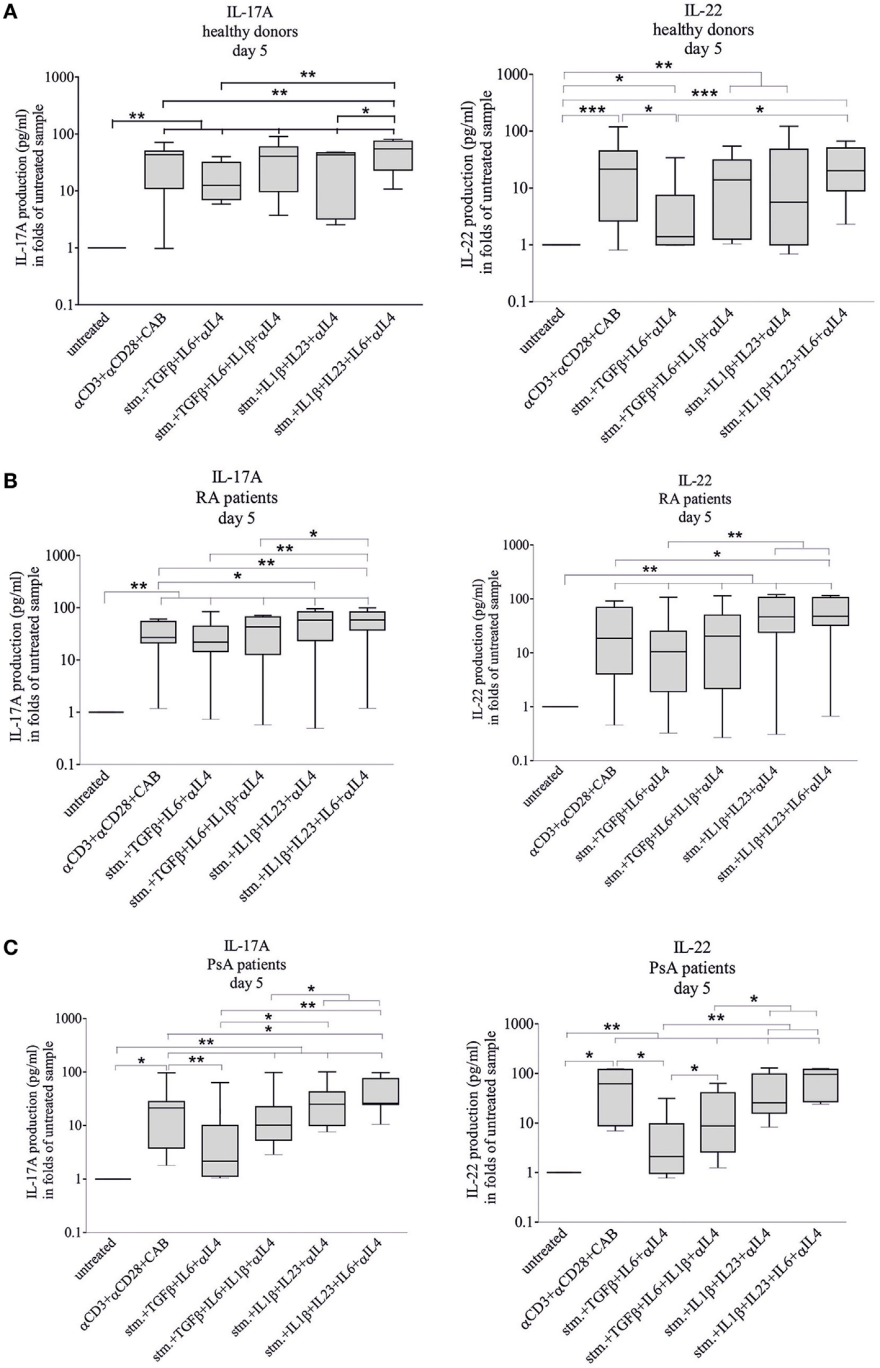
Interleukin-17A (IL-17A) and IL-22 cytokine secretion during differentiation. IL-17A and IL-22 cytokine secretions were measured during T-helper 17 differentiation of naive healthy- [**(A)** *n* = 12], rheumatoid arthritis (RA)- [**(B)** *n* = 9], psoriatic arthritis (PsA) patient-derived [**(C)** *n* = 7] CD4^+^ T cells. Naive CD4^+^ cells were stimulated and treated with cytokines as described earlier. The IL-17A and IL-22 levels were measured by enzyme linked immunosorbent assay method. Untreated cells on fifth day served as controls; the data are shown in a logarithmic scale. Median, minimum, and maximum values were indicated (Friedman test and pairwise Wilcoxon signed rank test, *p < 0.05, **p < 0.01, and ***p < 0.001).

Transcription factor (*RORC* and *TBX21*) expression and cytokine production (IL-17A and IL-22) were measured at the fifth day of the differentiation (Figures 4 and 5). At the fifth day, the stimulation (CD3/CD28/CAB) induced both *RORC* (*p* < 0.01) and *TBX21* (*p* < 0.01) gene expression of healthy-derived T cells (Figure 4A). *TBX21* mRNA did not change significantly neither in RA nor in PsA; while the *RORC* was upregulated in PsA (*p* < 0.05) but not in RA (Figures 4B,C).

The *RORC* mRNA of healthy CD4^+^ T cells was upregulated by all four cytokine combinations (*p* < 0.01, in all cases) (Figure 4A). The *RORC* expression of RA T cells was induced by TGF_β_ + IL1_β_ + IL6 and IL1_β_ + IL23 (*p* < 0.01 and *p* < 0.01, respectively) (Figure 4B); while in case of the PsA T lymphocytes, the effective cytokine combinations were TGF_β_ + IL6 and TGF_β_ + IL1_β_ + IL6 (*p* < 0.05 and *p* < 0.05, respectively) (Figure 4C). Importantly, *TBX21* mRNA was not upregulated by any cytokine combinations used either in RA or in PsA T cells, while the IL1_β_ + IL23 and IL1_β_ + IL6 + IL23 treatments increased its expression of the healthy donor-derived T cells (*p* < 0.01 and *p* < 0.01). These data strongly suggest that the regulation of *RORC* and *TBX21* expression is markedly altered in both RA and PsA.

The distribution of CCR4, CCR6, and CXCR3 chemokine receptor-carrying cells was studied by flow cytometry (Figure S4 in Supplementary Material). Although with the exception of TGF_β_ + IL6 treatment, all cytokine combinations altered the proportion of the healthy CCR4 and CCR6 double positive cells, none of the treatments had any significant effect on the dispersion of the different chemokine expressing cell populations (Figure S4 in Supplementary Material). Similarly, no meaningful differences were found in the chemokine receptor expression neither in RA nor in PsA (Figure S4 in Supplementary Material).

### Characterization of IL-17A and IL-22 Production

Interleukin-17A and IL-22 levels were measured by ELISA in cell culture media of the differentiating CD4 cells. Most treatments induced a large-scale increase of the cytokine production (Figure 5). Supplementation of the stimulating media (CD3/CD28/CAB) with TGF_β_ + IL6 + anti-IL4 antibody attenuated the IL-22 production of healthy- and PsA-derived but not those of the RA-derived T cells (*p* < 0.05 and *p* < 0.05, respectively) and the IL-17A production of the PsA T cells (*p* < 0.01).

Considering the potential *in vivo* variation of the conditions of Th17 differentiation (44), IL-17A and IL-22 production of the differentiating CD4^+^ T cells upon CD3/CD28/CAB stimulation, all cytokine and anti-IL4 treatments were compared between healthy donors and patient groups (Figure S5 in Supplementary Material). The IL-17A production pattern profoundly differed between healthy donors versus RA (*p* = 0.0000026) and healthy donors versus PsA-derived (*p* = 0.0001) samples. Although the IL-22 production was similar in PsA and healthy (*p* = 0.58) CD4^+^ T cells, it was markedly different in RA compared to healthy (*p* = 0.000006) groups, across treatment conditions. Interestingly, the RA versus PsA IL-17A pattern was rather similar (*p* = 0.85). By contrast, the IL-22 production was apparently different (*p* = 0.001) between the two patient groups (Figure S5 in Supplementary Material) pointing to the different cytokine profile of the two diseases.

While the transcription factor expression was similar in the 5th and 10th days of differentiation, the IL-17A and the IL-22 secretion decreased significantly after 5 days. In addition, the proportion of CCR6^+^CCR4^+^ cells increased significantly between 5th and 10th days of differentiation, upon IL1_β_ + IL23 + IL6 + anti-IL4 treatment both in RA and PsA, but not in healthy donors’ samples (data not shown).

Based on the LDA analysis, the healthy, RA, and PsA groups can be separated from each other with 81% discriminative power and the IL1_β_ + IL23 + IL6 + anti-IL4 induced IL-22 production served as the determinant factor (Figure S2C in Supplementary Material).

### Laboratory and Clinical Measures Related to Th17 Differentiation

**Figure 6:**
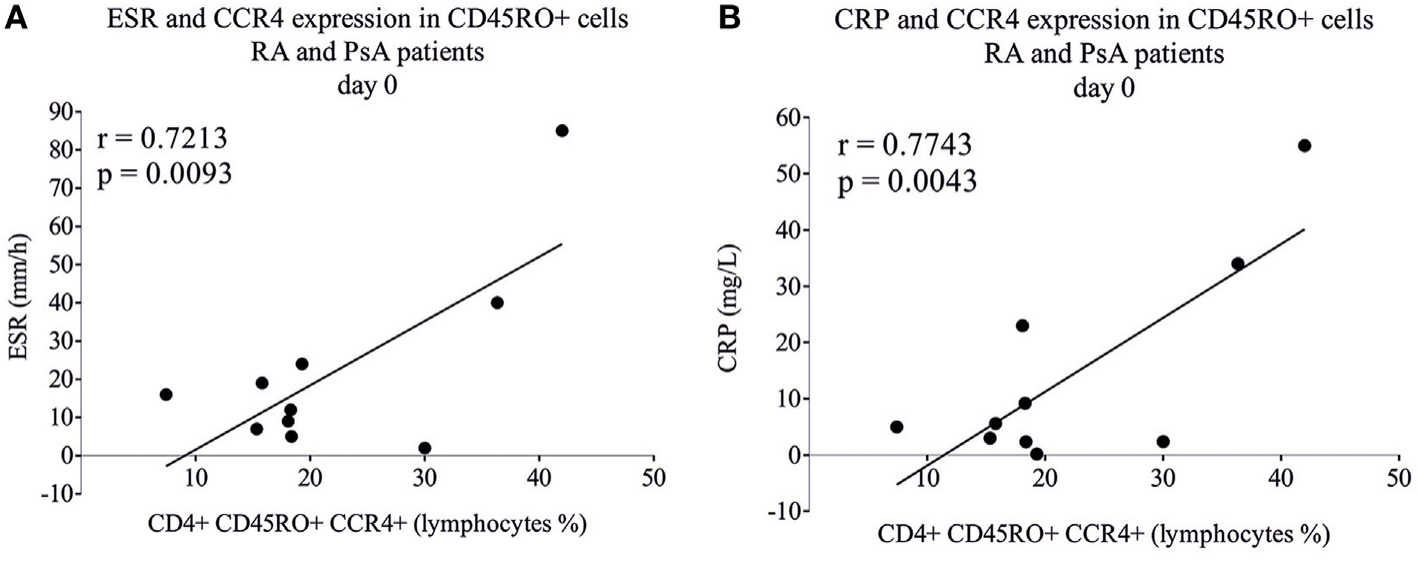
Correlation of inflammatory markers with chemokine receptor expression. CD4^+^CD45RO^+^ memory T cells were isolated by using magnetic separation and the CCR4 expression was measured by flow cytometry. Rheumatoid arthritis (RA) (*n* = 5) and psoriatic arthritis (PsA) patients’ (*n* = 5) samples were analyzed and correlated (Pearson correlation) to erythrocyte sedimentation rate (ESR) **(A)** and to C-reactive protein (CRP) **(B)**.

The number of CD4^+^CD45RO^+^CCR4^+^ lymphocytes of RA and PsA patients correlated strongly with the erythrocyte sedimentation rate (ESR) (*r* = 0.72, *p* = 0.0093) and C-reactive protein (CRP) (*r* = 0.77, *p* = 0.0043) (Figure 6), suggesting the proinflammatory role of these cells. No correlation was found between the disease activity, disease duration, medications used, and the baseline or induced *TBX21* or *RORC* expression. The IL-22 secretion of differentiating T cells (upon TGF_β_ + IL6 + IL1_β_ + anti-IL4 treatment) from leflunomide-treated RA patients’ was higher than those of the non-leflunomide-treated RA patients’ (*p* = 0.0159; data not shown).

## Discussion

Several lines of evidence support the crucial role of Th17-lymphocytes in inflammatory arthropathies (10, 11, 14, 33–35, 45–48). The main cytokines of human *in vitro* Th17 cell differentiation were described previously (2, 22, 25–30). However, the optimal conditions (cytokine concentrations, combinations, and treatment duration) are still elusive (26, 30, 48). Although numerous observations were published regarding the serum IL-17A levels and the frequency of Th17 cells, these data do not reflect the potential plasticity of the circulating naive T cells. Importantly, only few data have been published regarding the regulation of Th17 cell differentiation in inflammatory arthropathies (9, 11, 46, 48). Our present data confirm and extend previous observations regarding the role of key cytokines and transcription factors of the Th17 cell development in both RA and PsA (11, 48). Here, we show for the first time that naive CD4^+^CD45RO^−^ T lymphocytes are predisposed to differentiate into Th17 cells (based on the *RORC* expression); in addition, the *in vitro* Th17 cell development is significantly altered in both RA and PsA (Figure S6 in Supplementary Material). Interestingly, while the cytokine treatment-induced IL-17A production was profoundly different between RA and PsA as compared to healthy donors, the IL-22 production was different between RA and healthy donors, but not between healthy donors and PsA patients (Figure S5 in Supplementary Material).

Although the inductive role of IL-1_β_, IL-21, and IL-23 cytokines were described in RA- and PsA-derived T cells (11), according to our best knowledge, the most prominent cytokine combinations have not been studied systematically and compared in inflammatory arthropaties. Similarly to earlier observations in our hands, anti-IL4 antibody promoted Th17 differentiation (25). The precise role of TGF_β_ in Th17 development in immune-mediated diseases is still questionable (26, 28, 30, 48). In accordance with our present data, TGF_β_ treatment did not increase either the IL17 or the IL-22 secretion. Moreover, IL1_β_ + IL6 + IL23 cytokine treatment promoted IL-17A production in both healthy donors and in RA and PsA patients.

Higher *RORC* expression of CD4^+^ T cells was described in early RA and juvenile idiopathic arthritis but not in PsA. However, *RORC* expression of naive CD4 lymphocytes has not been investigated in these studies (11, 46). Elevated *RORC* and *TBX21* were measured in synovial fluid cells from PsA patients; although gene expression of naive and memory subpopulations were not determined in this work (49). In contrast to *RORC* expression, *TBX21* expression of naive and memory T cells strongly correlated to each other, indicating the independent regulation of the *RORC* in these cell types.

Human Th17 cells express chemokine receptors CCR4 and CCR6 (2, 24). Th22 cells express CCR4, CCR6, and CCR10; in addition, they produce IL-22 but not IL-17A (50, 51). Furthermore, Th22 cells express very low or undetectable levels of *RORC* and *TBX21* while AHR plays a cardinal role in IL-22 production of Th22 cells (31, 50, 52). In accordance, differentiating CD4^+^ T cells from the AHR agonist leflunomide-treated RA patients produced significantly more IL-22 than patients who underwent biological therapy. Furthermore, the frequency of CD4^+^CD45RO^+^CCR4^+^ lymphocytes correlated strongly with both ESR and CRP pointing to the potential proinflammatory role of these cells, as described previously in lupus nephritis (53). CCR4^+^CXCR3^+^ cells represent a major population of synovial cells in PsA patients (54). According to our results, based mainly on the percentage of CCR4^+^CXCR3^+^ cells, of the healthy, RA and PsA groups could be discriminated from each other, pointing to the essential role of these cells in inflammation.

Previous data suggest that the CD4^+^ T cells are hyporesponsive for TCR/CD3 stimulation due to their activation and cytokine-induced, decreased CD3ξ-chain expression (55–57). Our present data show that T lymphocyte stimulation (CD3/CD28/CAB) induced both *RORC* and *TBX21* expression, and IL-17A and IL-22 production in healthy donors. By contrast, in RA, none of the transcription factors, and in PsA, only *RORC* was induced (possibly due to the increased baseline expression), while production of both cytokines was increased by stimulation. This supports that early activation and engagement of the naive T cells are associated with an altered cytokine network in both RA and PsA.

Peripheral PsA and RA might be clinically similar, although the pathogenesis of the two diseases are substantially different (58). While antibodies such as anti-cyclic citrullinated peptide antibody or rheumatoid factor are key players in RA, no specific autoantibodies were identified in PsA. Some cytokines (TNF_α_, IL-1_β_, IL-17A, IL-22, and IL-23) play a central role in the pathogenesis of both conditions and Th17 cells clearly have a crucial role in both diseases. The applied cytokine treatments cause a distinct and characteristic change in transcription factors (*RORC* and *TBX21*) and cytokine production (IL-17A and IL-22) of healthy donors, RA, and PsA patients (Figures 4 and 5; Figure S5 in Supplementary Material). The complex effect of the cytokine combinations and their comparison of IL-17A and IL-22 production in RA and PsA were first described in this study, and these data support the different cytokine profile of the two diseases.

There are some limitations of our study, such as the small number of patients or the lack of synovial samples. Further work with higher number of patients are needed to study the naive T cells’ capacity to differentiate toward other T helper lineages as well, such as Th1, Th9, or Th22 (14, 59–62).

Our present data suggest in RA and PsA an early commitment of CD4^+^ T lymphocytes toward the Th17 lineage and a characteristic IL-17A and IL-22 production. These data may contribute to the understanding of Th17 differentiation both under physiological conditions and in RA and PsA. Further studies are needed to identify the underlying factors of the altered CD4^+^ T cell development in inflammatory arthropathies.

## Ethics Statement

The national and institutional ethics committees approved the study; informed consent was obtained from each individual [approval number: 21434-1/2016/EKU (593/16)].

## Author Contributions

EB, EIB, and GN designed the experiments; EB, NM, PK, OTK, EL, LK, and BÉ done the experimental work. BR and GN provided the clinical samples; EB, GN, and IKS did the statistical analysis. All authors were involved in data analysis and in drafting the article, all authors approved the final version to be published. GN had full access to all of the data in the study and takes responsibility for the integrity of the data and the accuracy of the data analysis.

## Conflict of Interest Statement

The authors declare that the research was conducted in the absence of any commercial or financial relationships that could be construed as a potential conflict of interest.

## Supplementary Material

Figure S1 Flow cytometry cell gating strategies. The naive (CD3^+^CD4^+^CD45RO^−^CD45RA^+^CD197^+^), effector (CD3^+^CD4^+^CD45RO^+^ CD45RA^−^CD197^−^/Tem), and central (CD3^+^CD4^+^CD45RO^+^CD45RA^−^CD197^+^/Tcm) memory T cells’ RAR-related orphan receptor gamma (RORγ) and T cell-specific T-box transcription factor T-bet (Tbet) transcription factor expression were studied. Isotype controls were indicated by blue and red shows the specific stainings. **(A–H)** and gates: R1-7: **(A)** SSC/FSC dot plot lymphocytes were gated (R1) from peripherial blood mononuclear cell (PBMC); **(B)** within R1 gate, CD3/CD4 dot plot the double-positive helper T cells were also gated (R2); **(C)** within R2 gate, CD45RA^+^ (R3); CD45RO^+^ (R4); **(D)** within R3 gate, the cells were more than 98% positive for CD197 (R5); **(E)** within R4 gate, discrimination of effector (R6) and central (R7) memory cells based on CD197 expression; **(F)** within R5 gate, RORγ and Tbet expression; **(G)** within R6 gate, RORγ and Tbet expression; **(H)** within R7 gate, RORγ and Tbet expression; Q1: Tbet positive cells; Q2: RORγ/Tbet positive cells; Q3: RORγ positive cells. **(J–M)** and gates: G1-G3: **(J)** SSC/FSC dot plot CD4^+^CD45RO^−^ lymphocytes were gated (G1) from magnetically separated cells; **(K)** within G1 gate, C-C chemokine receptor 6 (CCR6)^−^ (G2); CCR6^+^ (G3) cells were gated; **(L)** within G2 gate, CCR4 and C-X-C motif chemokine receptor 3 (CXCR3) expression; **(M)** within G3 gate, CCR4 and CXCR3 expression; Q1: CCR4 positive cells; Q2: CCR4/CXCR3 positive cells; Q3: CXCR3 positive cells I; **(I,N)** cell counts of different gates.

Figure S2 Discriminative power of the expression of transcription factors, chemokine receptors, and the cytokine production. Linear discriminant analysis based on the transcription factors **(A)**, chemokine receptor expressions **(B)**, and cytokine productions **(C)** in healthy, rheumatoid arthritis (RA), and psoriatic arthritis (PsA) groups.

Figure S3 CCR6^+^CCR4^+^CXCR3^+^, CCR4^+^CXCR3^+^, CCR4^+^, and CCR6^+^ chemokine receptor expression. The chemokine receptor expression of CD4^+^CD45RO^−^ naive and CD4^+^CD45RO^+^ memory T cells were studied by flow cytometry. Healthy volunteers’ [**(A)** *n* = 6], rheumatoid arthritis (RA) [**(B)** *n* = 5], and psoriatic arthritis (PsA) patients’ [**(C)** *n* = 5] data. The values are shown in a linear scale; the median, minimum, and maximum values are indicated (Wilcoxon signed rank test **p* < 0.05).

Figure S4 CCR6^+^CCR4^+^, CCR6^+^CXCR3^+^, and CXCR3^+^chemokine receptor expression during differentiation. Healthy donor- [**(A)** *n* = 6], rheumatoid arthritis (RA)- [**(B)** *n* = 5], and psoriatic arthritis (PsA)- [**(C)** *n* = 5] derived CD4^+^CD45RO^−^ naive T cells were stimulated and treated with different cytokine combinations to promote T-helper 17 cell differentiation from naive T cells as described earlier. Chemokine receptor expression was measured by flow cytometry on the fifth day. Relative chemokine receptor expression levels (compared to the naive untreated cells) are shown in a logarithmic scale; median, minimum, and maximum values of the normalized parameters were indicated (Friedman test and pairwise Wilcoxon signed rank test, **p* < 0.05).

Figure S5 Interrelation of IL-17A and IL-22 cytokine secretion during differentiation. IL-17A **(A,B)** and IL-22 **(C,D)** cytokine productions during T-helper 17 (Th17) differentiation of naive CD4^+^ T cells. Naive CD4^+^ T cells were stimulated and treated with cytokines as described earlier to promote Th17 differentiation. The IL-17A and IL-22 levels of cells from healthy donors’ (*n* = 12), rheumatoid arthritis (RA) (*n* = 9), and psoriatic arthritis (PsA) patients (*n* = 7) were measured by enzyme linked immunosorbent assay method on the fifth day of the differentiation. Interdependencies were evaluated by analysis of variance and by Tukey HSD tests. The differences between healthy, RA, and PsA groups of IL-17A **(A)** and IL-22 **(C)** levels by the effect of applied cytokine treatments were indicated.

Figure S6 Schematic representation of T-helper 17 differentiation. In addition to the IL-17A and IL-22 secretions during differentiation, the baseline and the cytokine treatment-induced transcription factor and chemokine receptor expression are distinct in both rheumatoid arthritis (RA) and psoriatic arthritis (PsA).

## Abbreviations

ACPA, anti-cyclic citrullinated peptide antibody; ACR, American College of Rheumatology; AOV, analysis of variance; CAB, F(ab′)2 fragment goat anti-mouse IgG; CCR6, C-C chemokine receptor 6; CD161, cluster of differentiation 161; cDNA, complementary deoxyribonucleic acid; CRP, C-reactive protein; CXCR3, C-X-C motif chemokine receptor 3; ELISA, enzyme linked immunosorbent assay; ESR, the erythrocyte sedimentation rate; EULAR, European League Against Rheumatism; FACS, fluorescence-activated cell sorting; FBS, fetal bovine serum; G-CSF, granulocyte colony-stimulating factor; GM-CSF, granulocyte-monocyte colony-stimulating factor; IBD, inflammatory bowel diseases; IFN_γ_, interferon gamma; IL-17A, interleukin-17; LDA, linear discriminant analysis; MACS, magnetic-activated cell sorting; MMP, matrix metalloproteinases; mRNA, messenger ribonucleic acid; MS, multiple sclerosis; NO, nitric oxide; *HPRT-1*, hypoxanthine phosphoribosyltransferase 1; PBMC, peripherial blood mononuclear cell; PsA, psoriatic arthritis; RA, rheumatoid arthritis; *RORC2*, retinoic acid-related orphan receptor variant 2; RORγ, RAR-related orphan receptor gamma; RPMI, Roswell Park Memorial Institute; SLE, systemic lupus erythematosus; T-bet, T cell-specific T-box transcription factor T-bet; *TBX21*, T-box 21; TGF_β_, transforming grow factor beta; Th17, T-helper 17; TNF_α_, tumor necrosis factor alpha; Tukey HSD, Tukey’s honest significant difference test; RF, rheumatoid factor; qPCR, quantitative polymerase chain reaction.
