# Supplementary material for: Distinct In Vitro T-Helper 17 Differentiation Capacity of Peripheral Naive T Cells in Rheumatoid and Psoriatic Arthritis
Source: Front Immunol. 2018 Apr 4;9:606. doi: 10.3389/fimmu.2018.00606 (PMC5893718; doi:10.3389/fimmu.2018.00606)
Supplement: Supplementary file 1 [file table_1.docx]

| **Table S1_Donor charachteristics** | |  |  |
| --- | --- | --- | --- |
|  | **Healthy donors** | **RA patients** | **PsA patients** |
| **Number of donors** | 12 | 12 | 7 |
| **Gender: females; males** | 10; 2 | 10; 2 | 2; 5 |
| **Mean age (yrs)** | 45.67 ± 13.53 | 53.83 ± 12.81 | 48.58 ± 11.17 |
| **Mean disease duration (year)** | NA | 15 ± 14.20 | 11 ± 7.33 |
| **Biological DMARD (%)** | NA | 50% | 43% |
| **Synthetic DMARD (%)** | NA | 80% | 70% |
| **Steroid (%)** | NA | 50% | 14% |
| **Untreated (%)** | NA | 17% | - |
| **Mean DAS28** | NA | 3.48 ± 1.33 | 3.06 ± 1.94 |
| **Mean ESR (mm/h)** | ND | 22.35 | 14.80 |
| **Mean CRP (mg/l)** | ND | 7.71 | 9.41 |
| **Mean RF (IU/ml)** | NA | 172.44 | NA |
| **Mean ACPA (AU/ml)** | NA | 466.58 | NA |
| **ACPA negative (%)** | NA | 8% | NA |
|  |  |  |  |
| Abbreviations: ACPA=anti-citrullinated protein antibody, CRP=C reactive protein, DAS=disease activity score, DMARD=disease modifying antirheumatic drug, ESR=erythrocyte sedimentation rate, NA=not applicable, ND= not determined, RF=rheumatoid factor | | | |
