# Supplementary material for: Distinct In Vitro T-Helper 17 Differentiation Capacity of Peripheral Naive T Cells in Rheumatoid and Psoriatic Arthritis
Source: Front Immunol. 2018 Apr 4;9:606. doi: 10.3389/fimmu.2018.00606 (PMC5893718; doi:10.3389/fimmu.2018.00606)
Supplement: Supplementary file 2 [file data_sheet_1.docx]

**Linear discriminant analysis**

Fisher’s linear discriminant analysis (LDA) aims at finding directions in the parameter space, such that between-class separability is maximized and within-class variability is minimized on the given dataset(Webb, 2002). Each direction is a linear combination of the original parameters characterized by a corresponding vector. As an important requirement, the vectors have to be of unit length and perpendicular to each other. These vectors are obtained as the solution of an eigenvector-type equation involving the within-group and the between-group covariance matrices. The obtained vectors can be sorted according to their importance given by the corresponding eigenvalues. Let $a_{1}$ denote the first vector, i.e. the one that maximizes between-class separability and minimizes within-class variability. Let $a_{2}$ denote the vector that maximizes the separation criterion subject to the constraint of being perpendicular to $a_{1}$. Let $a_{3}$ denote the vector that again optimizes the above objective, while being perpendicular to both $a_{1}$ and $a_{2}$, and continue similarly with $a_{4},a_{5}$, etc. Let $k$ denote the number of classes, and $p$ the number of parameters. In total, no more than $k-1$ of such vectors can be obtained. Also, naturally, no more than $p$ vectors can be obtained, thus leading to at most $\min\left( k-1,p \right)$ vectors. Let X denote the ($n\times p$) data matrix with all the $n$ observations from the $p$ variables. ${LD}_{1}={Xa}_{1}$ then gives the coordinates of the original observations in the direction given by $a_{1}$. Similarly define ${LD}_{2}={Xa}_{2}$, etc. If ${LD}_{1}$ is plotted against ${LD}_{2}$, we can visualize the separation of the groups in two dimensions (provided that there are at least three groups), while using ${LD}_{3}$ in addition leads to the possibility of a three-dimensional visualization (provided that there are at least four groups). According to the choice of $a_{1},a_{2},a_{3}$, this is the best possible two/three-dimensional separation-plot of the data.

Furthermore, one can define linear discriminant functions using these vectors $a_{i}$in order to obtain a decision rule for classifying new observations into one of the groups. A commonly used approach is a nearest-mean type classifier in the transformed space, i.e. a new observation gets the predicted label of the group-mean that lies closest to it in the transformed space. This procedure is actually optimal if the data are multivariate normal in each class and the covariance matrix is the same for all classes.

Webb AR. Statistical Pattern Recognition, Second Edition. John Wiley & Sons Ltd., Chichester, England. (2002)
