# Supplementary figures and images for: Distinct In Vitro T-Helper 17 Differentiation Capacity of Peripheral Naive T Cells in Rheumatoid and Psoriatic Arthritis
Source: Front Immunol. 2018 Apr 4;9:606. doi: 10.3389/fimmu.2018.00606 (PMC5893718; doi:10.3389/fimmu.2018.00606)

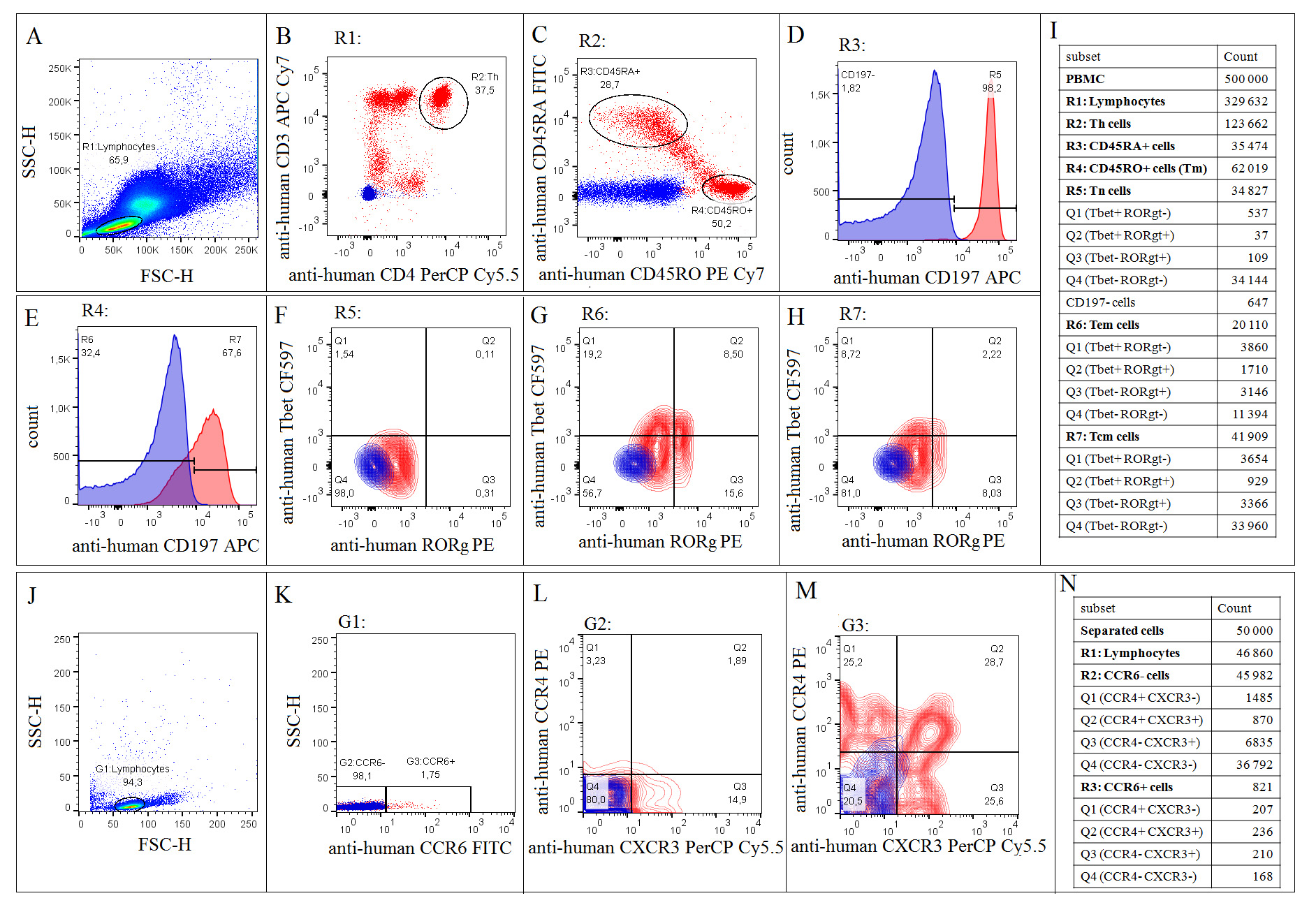

Supplement: Figure S1 — Flow cytometry cell gating strategies. The naive (CD3+CD4+CD45RO−CD45RA+CD197+), effector (CD3+CD4+CD45RO+ CD45RA−CD197−/Tem), and central (CD3+CD4+CD45RO+CD45RA−CD197+/Tcm) memory T cells’ RAR-related orphan receptor gamma (RORγ) and T cell-specific T-box transcription factor T-bet (Tbet) transcription factor expression were studied. Isotype controls were indicated by blue and red shows the specific stainings. (A–H) and gates: R1-7: (A) SSC/FSC dot plot lymphocytes were gated (R1) from peripherial blood mononuclear cell (PBMC); (B) within R1 gate, CD3/CD4 dot plot the double-positive helper T cells were also gated (R2); (C) within R2 gate, CD45RA+ (R3); CD45RO+ (R4); (D) within R3 gate, the cells were more than 98% positive for CD197 (R5); (E) within R4 gate, discrimination of effector (R6) and central (R7) memory cells based on CD197 expression; (F) within R5 gate, RORγ and Tbet expression; (G) within R6 gate, RORγ and Tbet expression; (H) within R7 gate, RORγ and Tbet expression; Q1: Tbet positive cells; Q2: RORγ/Tbet positive cells; Q3: RORγ positive cells. (J–M) and gates: G1-G3: (J) SSC/FSC dot plot CD4+CD45RO− lymphocytes were gated (G1) from magnetically separated cells; (K) within G1 gate, C-C chemokine receptor 6 (CCR6)− (G2); CCR6+ (G3) cells were gated; (L) within G2 gate, CCR4 and C-X-C motif chemokine receptor 3 (CXCR3) expression; (M) within G3 gate, CCR4 and CXCR3 expression; Q1: CCR4 positive cells; Q2: CCR4/CXCR3 positive cells; Q3: CXCR3 positive cells I; (I,N) cell counts of different gates. [file image_1.jpeg]

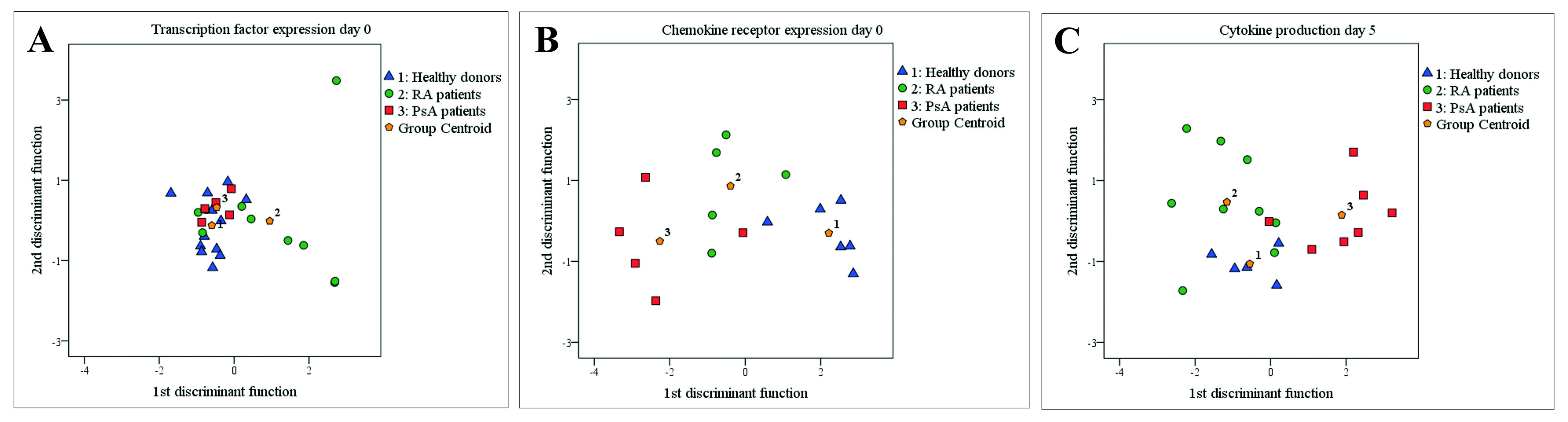

Supplement: Figure S2 — Discriminative power of the expression of transcription factors, chemokine receptors, and the cytokine production. Linear discriminant analysis based on the transcription factors (A), chemokine receptor expressions (B), and cytokine productions (C) in healthy, rheumatoid arthritis (RA), and psoriatic arthritis (PsA) groups. [file image_2.jpeg]

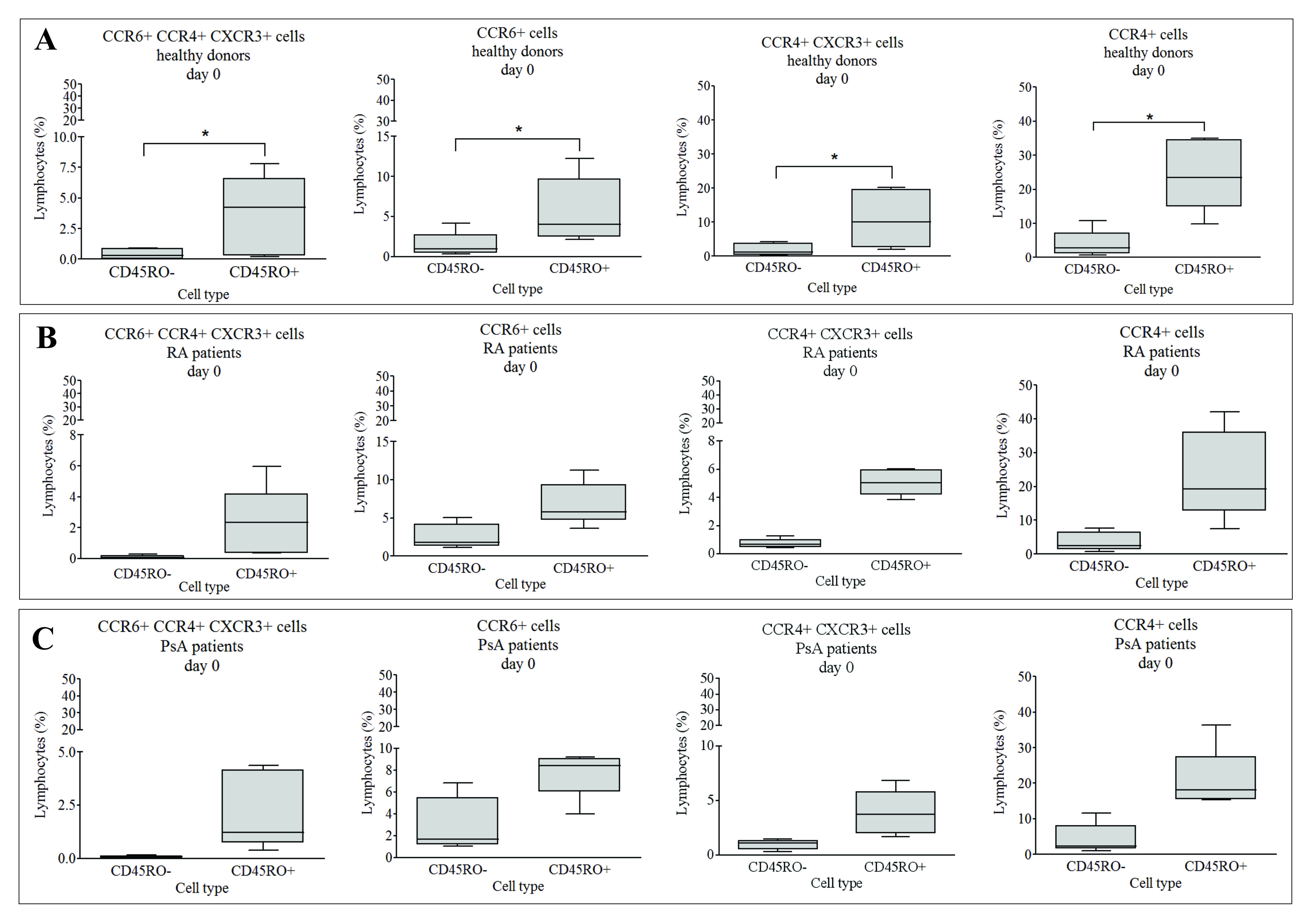

Supplement: Figure S3 — CCR6+CCR4+CXCR3+, CCR4+CXCR3+, CCR4+, and CCR6+ chemokine receptor expression. The chemokine receptor expression of CD4+CD45RO− naive and CD4+CD45RO+ memory T cells were studied by flow cytometry. Healthy volunteers’ [(A) n = 6], rheumatoid arthritis (RA) [(B) n = 5], and psoriatic arthritis (PsA) patients’ [(C) n = 5] data. The values are shown in a linear scale; the median, minimum, and maximum values are indicated (Wilcoxon signed rank test *p < 0.05). [file image_3.jpeg]

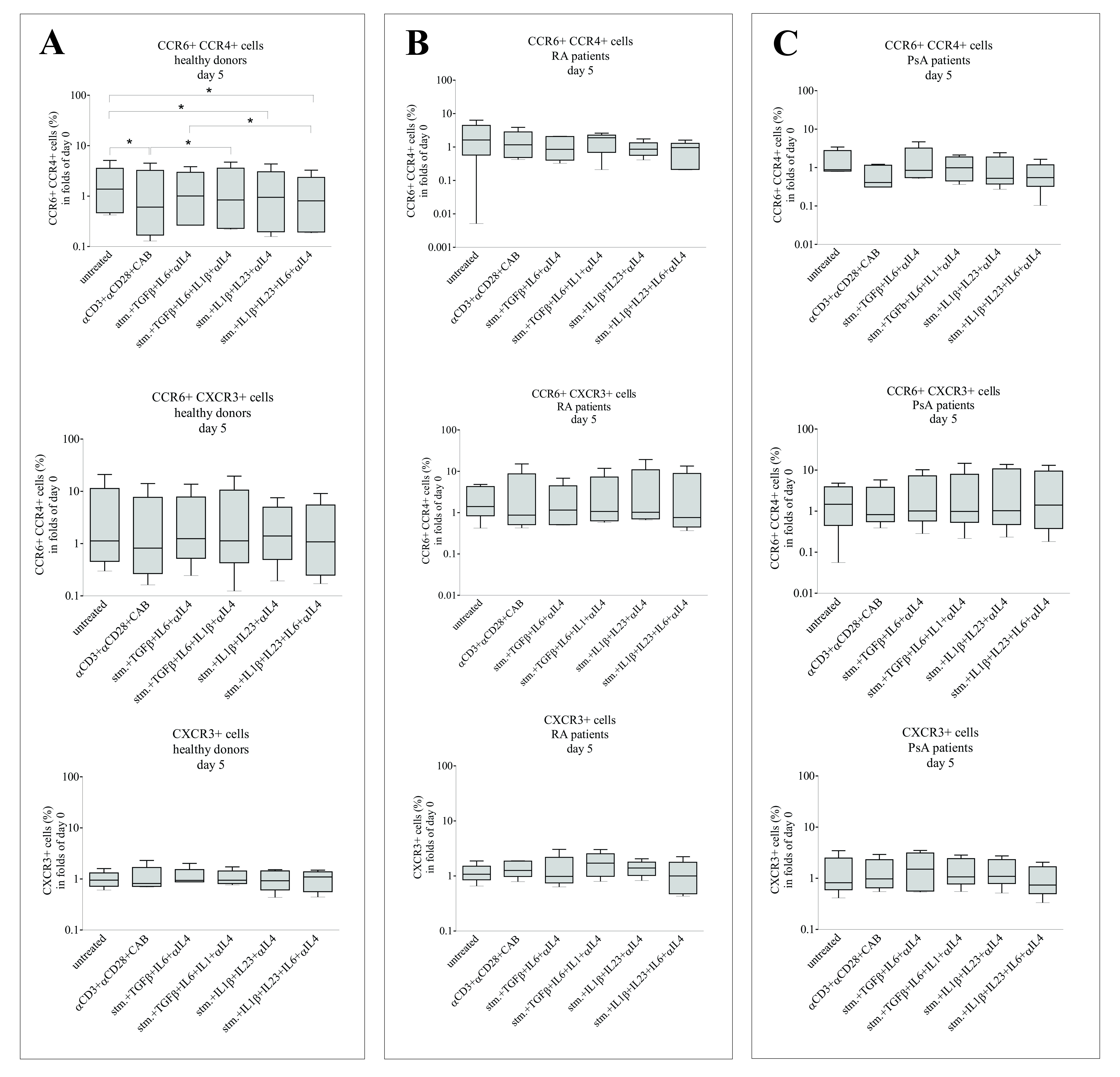

Supplement: Figure S4 — CCR6+CCR4+, CCR6+CXCR3+, and CXCR3+chemokine receptor expression during differentiation. Healthy donor- [(A) n = 6], rheumatoid arthritis (RA)- [(B) n = 5], and psoriatic arthritis (PsA)- [(C) n = 5] derived CD4+CD45RO− naive T cells were stimulated and treated with different cytokine combinations to promote T-helper 17 cell differentiation from naive T cells as described earlier. Chemokine receptor expression was measured by flow cytometry on the fifth day. Relative chemokine receptor expression levels (compared to the naive untreated cells) are shown in a logarithmic scale; median, minimum, and maximum values of the normalized parameters were indicated (Friedman test and pairwise Wilcoxon signed rank test, *p < 0.05). [file image_4.jpeg]

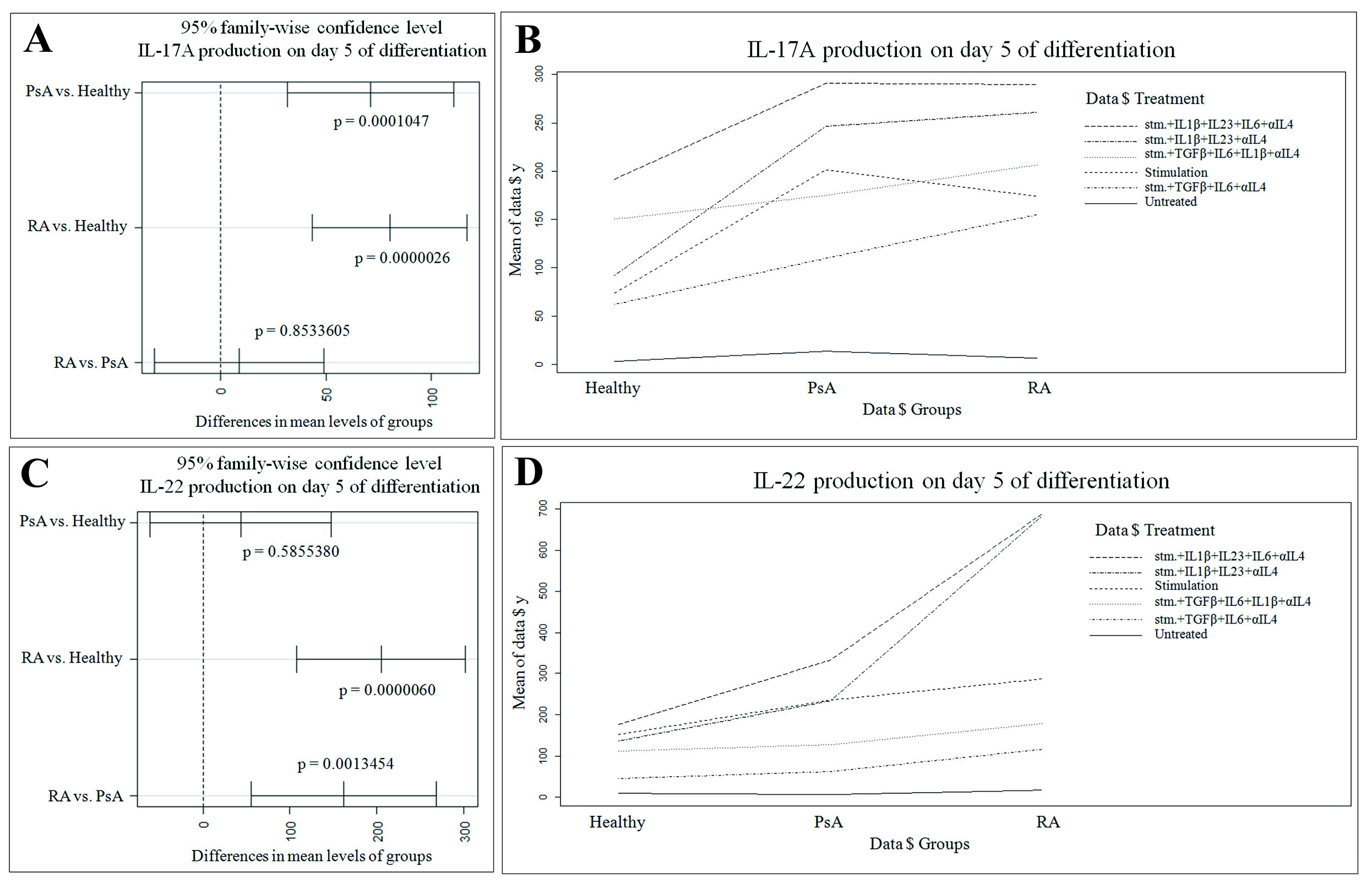

Supplement: Figure S5 — Interrelation of IL-17A and IL-22 cytokine secretion during differentiation. IL-17A (A,B) and IL-22 (C,D) cytokine productions during T-helper 17 (Th17) differentiation of naive CD4+ T cells. Naive CD4+ T cells were stimulated and treated with cytokines as described earlier to promote Th17 differentiation. The IL-17A and IL-22 levels of cells from healthy donors’ (n = 12), rheumatoid arthritis (RA) (n = 9), and psoriatic arthritis (PsA) patients (n = 7) were measured by enzyme linked immunosorbent assay method on the fifth day of the differentiation. Interdependencies were evaluated by analysis of variance and by Tukey HSD tests. The differences between healthy, RA, and PsA groups of IL-17A (A) and IL-22 (C) levels by the effect of applied cytokine treatments were indicated. [file image_5.jpg]

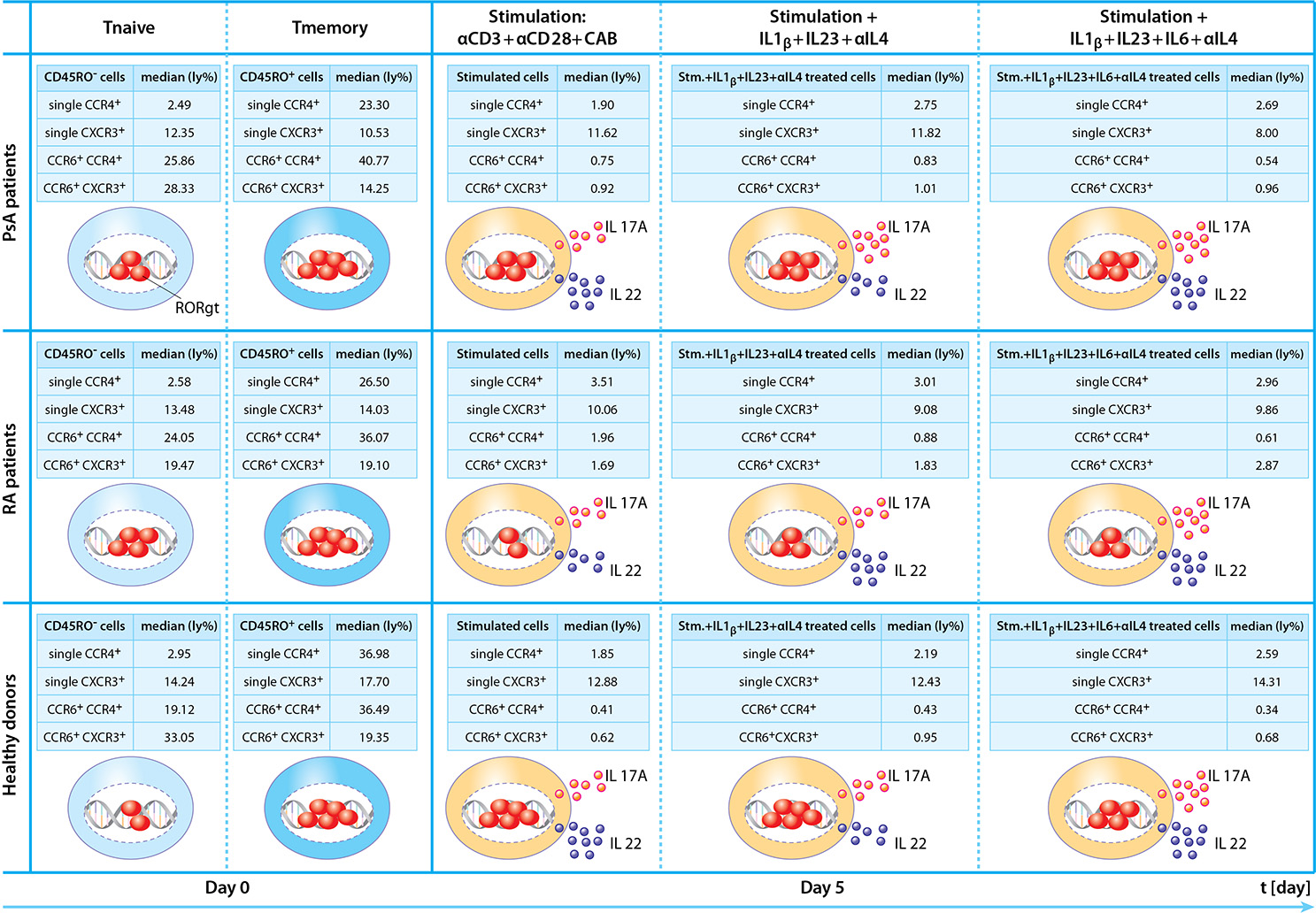

Supplement: Figure S6 — Schematic representation of T-helper 17 differentiation. In addition to the IL-17A and IL-22 secretions during differentiation, the baseline and the cytokine treatment-induced transcription factor and chemokine receptor expression are distinct in both rheumatoid arthritis (RA) and psoriatic arthritis (PsA). [file image_6.jpeg]
